# Emerging Horizons in Plant Genetics and Breeding

**DOI:** 10.3390/ijms241411621

**Published:** 2023-07-19

**Authors:** Manosh Kumar Biswas

**Affiliations:** Department of Genetics and Genome Biology, University of Leicester, Leicester LE1 7RH, UK; manosh24@yahoo.com; Tel.: +44-(0)774-118-9203

Plant genetics and breeding have made significant progress in recent years, especially with the emergence of genomics. The availability of cost-effective Next-Generation Sequencing (NGS) technology has enabled the identification and characterization of novel genes, leading to a better understanding of the genetic foundation of plant traits and the development of novel genetic tools and methods. Furthermore, genome editing has the potential to revolutionize plant breeding by producing crop varieties with improved yield, quality, and resistance to biotic and abiotic stresses.

This Special Issue features papers that present new and exciting research results applicable to plant breeding and genetics, with a focus on improving plants. The articles cover a diverse range of topics within Genetics and Breeding research, including gene identification and characterizations for various plant species (such as R2R3-MYB in *Ananas* spp., NPF family genes in *Brassica rapa*, 2-Acetyl-1-pyrroline gene in soybeans, BaZFP1 in moss, KFB family in moso bamboo, and 14-3-3 gene family in bamboo). Additionally, the papers cover areas such as transcriptomics, molecular breeding, and genome editing. The next section provides a brief overview of each published article.

In plant sciences, gene identification and functional characterization are crucial for enhancing plant breeding programs, developing new crop varieties with desired traits, and improving our understanding of fundamental biological processes in plants.

Wei et al. [1] conducted a comprehensive bioinformatic analysis of the R2R3-MYB gene family in *Ananas comosus* var. bracteatus to identify and characterize the MYB gene family. They identified 99 R2R3-MYB genes and classified them into 33 subfamilies using phylogenetic analysis. Gene duplication and collinearity analysis revealed that segmental duplication was responsible for amplifying the AbR2R3-MYB gene family. Promoter analysis showed that AbR2R3-MYB genes may play important roles in hormone-induced anthocyanin biosynthesis. Tissue-specific expression patterns of 10 AbR2R3-MYB genes suggest that they may regulate anthocyanin biosynthesis in the plant’s flower, leaf, and bract. Overall, this study provides valuable insights into the mechanism underlying anthocyanin regulation in *A. comosus* var. bracteatus.

Yang et al. [2] discovered 85 NPF genes in *Brassica rapa*, which were classified into eight subfamilies. The study found that these genes were conserved throughout the genus and that their expansion was due to tandem duplication. Transcriptomic analysis showed that specific NPF genes played important roles in pollen development and nitrate absorption, while others responded to vernalization and clubroot disease. These findings suggest that NPF genes have the potential for genetic improvement studies of *B. rapa*.

Aroma is an essential quality of vegetable soybeans for their market value; the 2-acetyl-1-pyrroline (2AP) compound is responsible for aroma formation, but it is unclear how it differs in aromatic and non-aromatic soybeans. Zhao et al. [3] explored the differences in aroma formation pathways between aromatic and non-aromatic vegetable soybeans by analyzing metabolomics and gene expression. The results show that the differentially accumulated metabolites (DAMs) in the two varieties contributed to their phenotypic differences. The synthesis pathway of 2-acetyl-1-pyrroline (2AP) was mainly influenced by the differences in amino acids and derivatives, with 1-pyrroline-5-carboxylate (P5C) being the main precursor for 2AP synthesis. The associated synthetic genes were verified, and the betaine aldehyde dehydrogenase 2 (GmBADH2) mutant was found to be crucial for 2AP synthesis and the synthesis of 4-aminobutyric acid (GABA) in vegetable soybean.

BaZFP1 is an important C2H2 zinc finger protein subfamily gene in plants, and it plays an important role in regulating growth, development, and responses to abiotic stress. Zhou et al. (2023) [4] identified 57 BaZFP transcripts in desiccation-tolerant moss and classified them into four groups. BaZFP1 is a nuclear C2H2-ZFP that acts as a positive regulator of growth and development in both moss and *Arabidopsis thaliana*. Zhou et al. reported that the overexpression of BaZFP1 promotes differentiation and growth of gametophytes and regulates the whole growth and development cycle in Arabidopsis. Moreover, the quantitative analysis of genes related to growth and development in transgenic moss and Arabidopsis shows significant changes in their expression levels.

Kelch repeat F-box (KFB) proteins are a group of E3 ubiquitin ligases that play a vital role in regulating various biological processes in plants, including hormone signalling, circadian rhythms, and responses to biotic and abiotic stresses. KFBs recognize specific target proteins and facilitate their ubiquitination, leading to their degradation by the 26S proteasome, which is vital for maintaining protein homeostasis and controlling protein levels involved in different cellular processes. Yang et al. [5] identified and characterized 19 PeKFBs in moso bamboo with F-box and Kelch domains that were unevenly distributed across 12 chromosomes and classified into eight subclades. The tissue-specific gene expression analysis revealed that PeKFBs were expressed differentially in various tissues of moso bamboo and under drought and cold stresses. The promoters of all PeKFBs contained stress-related cis-elements, and PeKFB9 was found to interact with PeSKP1-like-1 and PePRX72-1, regulating the activity of peroxidase in proteolytic turnover. The study provides a foundation for understanding the function and regulation of PeKFBs in moso bamboo, particularly in response to stress, and for exploring the molecular mechanism of PeKFB9 in regulating lignin polymerization by degrading peroxidase.

The 14-3-3 gene family is a highly conserved group of regulatory proteins that play important roles in various cellular processes in plants, including cell cycle control, signal transduction, metabolism, stress responses, and protein trafficking. These proteins interact with a wide range of target proteins, often via phosphorylation-dependent mechanisms, to modulate their activity, localization, and stability. Gou et al. [6] identified and characterized the 14-3-3 gene family in three bamboo species, identifying 58 genes that were grouped into ε and non-ε groups based on phylogenetic analysis. The bamboo 14-3-3 genes had diverse structures and motifs, and collinearity analysis suggested purification selection during evolution. Tissue-specific expression analysis revealed functional diversity during growth and development. Co-expression analysis and yeast two-hybrid assays showed that Pe14-3-3b/d interacted with ribosomal genes and participated in shoot growth and development. Overexpression of Pe14-3-3b in Arabidopsis promoted growth, indicating its potential for improving bamboo.

Transcriptomics provides information about which genes are active and how they are regulated in a particular biological system. This information is particularly valuable in plant breeding research, where it can be used to identify genes that are associated with desirable traits such as disease resistance, drought tolerance, and increased yield. By comparing the transcriptomes of different plant varieties or lines, researchers can identify differentially expressed genes associated with desirable traits. This information can be used to develop molecular markers that are used in breeding programs to select plants with desired traits. In this Special Issue, some of the articles present transcriptome data mining and utilize them for gene identification and functional characterizations. We describe them briefly in the next few paragraphs.

Abiotic stressors like drought and salinity seriously hamper the growth and development of plants; consequently, it is important to understand drought and salinity resistance mechanisms to increase cotton production under drought and salt conditions. Chen et al. [7] identified 8806 and 9108 differentially expressed genes (DEGs) from transcriptome data mining of PEG-induced osmotic and salt stress in cotton. They demonstrated that the hub gene GhWRKY46 could alter salt and PEG-induced drought resistance in cotton through the virus-induced gene silencing (VIGS) method.

Seed size is one of the key agronomic traits for yield in peanut (*Arachis hypogaea* L.) breeding. Until now, very little was known about the regulation mechanism of peanut seed size. In their study, Guo et al. [8] used transcriptomic methods to understand the molecular mechanism of peanut seed size and identify the key gene that controls peanut yield and quality. They identified a *small seed mutant1* (*ssm1*) by irradiating peanut cultivar Luhua11 (LH11) using a ^60^Coγ ray.

Anthocyanins are known to be responsible for the colours of many plant species. In a study by Niu et al., RNA-seq data were generated from different fruit developmental stages of kiwifruit (*Actinidia arguta* var. purpurea), which were subsequently analyzed to understand the molecular mechanisms involved in anthocyanin biosynthesis during *A. arguta* fruit development.

Long-term seed storage can lead to a deterioration in viability. To understand the mechanisms behind this, Pereira Neto et al. applied transcriptomic tools to investigate the deterioration of viability in *A. fraxinifolium* Schott accessions caused by long-term storage. The result showed that various genes were expressed differently, such as genes for late embryogenesis abundant (LEA) proteins, genes involved in the photosystem, genes that produce glycine-rich proteins (GRP), and many transcription factors related to embryo development and ubiquitin-conjugating enzymes. These findings enhance our understanding of the genes associated with the aging of seeds and can be used as a foundation for further characterizing the functional aspects of the seed aging process in *A. fraxinifolium*.

Comparative transcriptomics is a popular method for identifying disease-resistant genes. Qin et al. [9] utilized this method to identify the *Pseudomonas syringae* pv. actinidiae (Psa) resistant gene in kiwifruit. Their study compared the transcriptome sequencing of the resistant kiwifruit cultivar Maohua (MH) and the susceptible cultivar Hongyang (HY) after infection with Psa. Subsequently, differential expression gene (DEG) analysis methods were applied to identify candidate genes.

Molecular breeding, a cutting-edge approach in modern plant breeding, has revolutionized the field by providing powerful tools for genetic analysis and manipulation of important traits in crops. This technique allows plant scientists to identify favorable traits at the molecular level rather than having to rely solely on observable traits that can be influenced by environmental conditions. The genome-wide association study (GWAS) has emerged as a significant method for identifying QTL (quantitative trait loci) and genomic regions in various species. In plants, RNA-seq has been extensively utilized to uncover the causal genes in GWAS. In the context of rice breeding, the identification of genes associated with cold tolerance is crucial due to rice’s sensitivity to cold stress. The genetic variation present in Asian cultivated rice has proven to be a valuable resource for discovering genetic loci and cold-related genes. Li et al. [10] conducted a GWAS study on 338 natural rice cultivars, successfully identifying 235 significantly associated single-nucleotide polymorphisms. Within these associations, 11 quantitative trait loci were linked to cold stress, while 12 were associated with chilling acclimation in seedlings. A total of 10 candidate genes were found within the cold stress-related QTLs, and 15 candidate genes were identified in the chilling acclimation-related QTLs. Notably, two novel candidate genes were discovered to be up-regulated during chilling acclimation, and a new gene encoding a putative E3 ubiquitin-protein ligase was found to be associated with cold tolerance.

In their study, Govta et al. [11] evaluated 232 wheat accessions from diverse eco-geographic regions under different water regimes (well watered and water-limited) and genotyped them using 71,571 SNPs. The goal was to identify genetic loci in the bread wheat genome that influence yield stability and quality under water stress. The analysis revealed 154 marker–trait associations (MTAs) that were highly significant for 9 out of 14 traits. Around these MTAs, there were 513 and 604 high-confidence genes on the *Triticum aestivum* cv. Chinese Spring genome, with 41 candidate genes potentially associated with abiotic stresses. The distribution of MTAs was found to be asymmetrical between the A- and B-genomes under water-limited conditions, suggesting differential contributions of homoeologous genes to various traits. Additionally, the study identified 20 MTAs on Chromosome 6B, including 9 QTLs, with 13 MTAs specifically associated with water-limited conditions. Furthermore, several QTLs on chromosomes 2B and 6B were linked to heat stress and the maintenance of thousand-grain weight under high temperatures. These findings have significant implications for breeding programs focused on improving drought and heat stress tolerance in wheat.

Genome editing in plants allows for the precise alteration of the genome sequence associated with important plant phenotypic traits. This technology enables direct improvement in elite breeding lines, targeting specific and crucial phenotypic traits. Previously, achieving such improvements required traditional crossing and backcrossing to generate a series of progenies and select the best line while removing residual genome from the donor line.

Plant cell walls are complex structures consisting of hemicellulose, pectin, cellulose microfibrils, proteins, and aromatic substances. Pectin methylesterase inhibitors (PMEI) have the ability to inhibit plant pectin methylesterase (PME), and the stability of the PMEI-PME combination is influenced by pH. Li et al. [12], in their study, 45 PMEI genes were identified from rice and analyzed their structure, motifs, cis-acting elements, chromosomal distribution, gene duplication, and phylogenetic relationship. The 45 OsPMEIs were categorized into three groups (I, II, and III) based on their phylogenetic tree and chromosomal distribution. Among them, OsPMEI12 was found on Chr3 and showed five pairs of gene duplication. To investigate the functions of OsPMEI, the CRISPR/Cas9 gene editing technology was employed to edit the OsPMEI12 gene, resulting in the creation of two mutant pmei12 lines. These lines were then used to study the impact of OsPMEI on plant growth, development, and their response to cadmium (Cd) stress.

PMEI plays a crucial role in plant cell wall extension and stress response. It modulates PME activity, leading to changes in plant methyl ester levels, which affect various aspects such as cell wall structure, pollen tube elongation, root development, and stress resistance. Overexpression of OsPMEI28 or AtPMEI5 leads to reduced plant height and deformed pods, highlighting the significance of PMEI in plant cell wall extension and stress response. This study utilized CRISPR/Cas9 gene editing to examine the impact of PMEI on rice growth and cell wall synthesis.

The European chestnut tree, Castanea sativa, is vulnerable to ink disease, chestnut blight, and the Asian gall wasp. Traditional breeding techniques face limitations in improving this tree due to heterozygosity, long generation times, and low transformation efficiency. The CRISPR/Cas9 system is being explored for gene editing in woody plants, although it faces challenges such as low transformation efficiency and difficulties in organogenesis [13]. By delivering the CRISPR/Cas9 system as ribonucleoproteins (RNPs), stable integration of recombinant DNA can be minimized or avoided. This RNP delivery method acts directly on the target site without requiring transcription activation. The researchers successfully applied the CRISPR/Cas9 system via RNPs in *C. sativa* protoplasts, targeting the phytoene desaturase (pds) gene involved in chlorophyll biosynthesis. They obtained transgene-free protoplasts and achieved regeneration. Further studies on regenerating genome-edited protoplasts hold the potential to develop DNA-free, genome-edited chestnut plants.
